# The New Hypnotic Medinal in Psychiatric Practice

**Published:** 1909-04

**Authors:** 


### ABSTRACTS.

### The New Hypnotic Medinal in Psychiatric Practice

Dr. S. Prato, (Insane Asylum of Ancona). (*Rivista Sper. di Fren.*, Vol. XXXIV, March 4, 1908), remarks that he administered medinal (sodium diethylbarbiturate) to about thirty insane subjects, most of them females, insomnia being more prevalent in that sex. Some received it continuously for about two months, without there being, after ten to twenty days, any material fluctuation in the period of sleep. Per os 7½ to 10 grains, dissolved in water or wine with sugar, were given three hours after the evening meal and generally readily taken. To excited or restless patients it was given rectally or subcutaneously. The results were satisfactory; the effect of this new hypnotic, which should soon meet with adoption is not only hypnotic but also sedative.

In old females no deleterious action on the heart could be seen. In a case of maniacal excitement with serious compensatory disturbances, medinal caused no exacerbation, nor did it counteract the beneficial effect of the cardiac remedies employed. Blood pressure, pulse and respiration were not affected by medinal. Never was headache or stomachache complained of, never was the least irritation noted after the numerous injections. Of course, the effect of the remedy is stronger or weaker in accordance with the condition of the patient.

Everyone knows the danger of the action of chloral on the heart, and the uncertainty of sulphonal, paraldehyd and trional. The advantages of veronal over these induced its wide acceptance. Medinal, because of its easier administration, reliability and harmlessness, should supplant veronal. It is always effective and of rapid and prolonged effect. The sleep attained with it closely resembles natural sleep, is quiet and non-disturbed. Awakening is not accompanied by headache, vertigo or malaise. The effect of medinal is not only hypnotic but also sedative. Continued use does not show cumulative by-effects. It retains its efficacy for a long time; accustomance to it is much slower than with other hypnotics. It can be given without hesitation to old and weakly individuals. Given per os it is well borne and easily absorbed in the intestine. The subcutaneous injection is effective and devoid of drawbacks.

